# Comparison of Functional Movement, Balance, Vertical Jumping, Hip Strength and Injury Risk in Adolescent Female Volleyball Players with and Without Chronic Ankle Instability

**DOI:** 10.3390/medicina61091547

**Published:** 2025-08-28

**Authors:** Abdullah Sinan Akoğlu, Rıdvan M. Adın, Ahmet Mustafa Ada, Volga Bayrakcı Tunay, Zafer Erden

**Affiliations:** 1 Department of Physiotherapy and Rehabilitation, Faculty of Health Sciences, Ordu University, 52200 Ordu, Turkey; fzt.sinanakoglu@gmail.com; 2 Department of Physical Fitness Test and Evaluation, Turkish Armed Forces Sport School, 06420 Ankara, Turkey; ahmetmustafaada@gmail.com; 3 Department of Sports Physiotherapy and Rehabilitation, Faculty of Physical Therapy and Rehabilitation, Hacettepe University, 06100 Ankara, Turkey; volgatunay@hacettepe.edu.tr; 4 Department of Musculoskeletal Physiotherapy and Rehabilitation, Faculty of Physical Therapy and Rehabilitation, Hacettepe University, 06100 Ankara, Turkey; zerden@hacettepe.edu.tr

**Keywords:** adolescent athlete, ankle injury, chronic ankle instability, functional movement screen, dynamic balance, hip strength, vertical jumping, injury risk

## Abstract

*Background and Objectives*: Chronic ankle instability (CAI), a prevalent injury among female volleyball players, can negatively affect functional performance and increase the risk of further injury. The aim of this study was to compare functional movement quality, dynamic balance, vertical jumping performance, hip muscle strength, and risk of injury between adolescent female volleyball players with unilateral CAI and those without CAI. *Materials and Methods*: This cross-sectional study included 46 adolescent female volleyball players, divided into CAI (*n* = 23) and control (*n* = 23) groups based on predefined criteria. Functional movement quality was assessed using the Functional Movement Screen (FMS), and dynamic balance was evaluated with the Y-Balance Test (YBT). Maximal isometric strength of the hip muscles (flexors, extensors, abductors, adductors, and internal and external rotators) was measured using hand-held dynamometry, and vertical jumping performance was assessed using countermovement jump tests. Injury risk was classified based on established cut-off values for the FMS-composite and YBT-anterior reach asymmetry scores. *Results*: The CAI group demonstrated significantly lower FMS-composite scores (*p* = 0.007), reduced anterior reach on the YBT (*p* = 0.004), and decreased strength in the hip flexors (*p* = 0.007) and hip adductors (*p* = 0.044), supported by moderate effect sizes. No significant group differences were observed in the other YBT directions, vertical jump tests, or the other hip muscles (*p* > 0.05). A greater proportion of athletes in the CAI group were classified as high risk for injury based on both FMS-composite (*p* = 0.022) and YBT-anterior reach asymmetry (*p* = 0.001) cut-off values, supported by moderate and relatively strong effect sizes, respectively. *Conclusions*: Adolescent female volleyball players with unilateral CAI showed impaired movement quality, balance deficits, hip muscle weakness, and increased injury risk. These results highlight the importance of targeted interventions and broader investigations into CAI in adolescent athletes.

## 1. Introduction

Volleyball is a highly dynamic sport characterized by movements that require speed, strength, and explosive power, such as lateral shuffling, vertical jumps, blocks, and attack hits [1]. Therefore, volleyball encompasses activities that place athletes at increased risk for ankle injuries [1,2]. Consistently, ankle injuries (particularly lateral ankle sprains) are the most common injuries among adolescent female volleyball players, comprising 25% to 36.6% of all volleyball-related injuries [3].

Ankle sprains are reported to occur more frequently in females than in males (13.6 vs. 6.94 per 1000 exposures), in adolescents compared to adults (1.94 vs. 0.72 per 1000 exposures) and in athletes relative to other populations [4]. Despite these facts, ankle sprains are often underestimated by adolescent athletes. This perception is further supported by the low number of athletes who adhere to preventive strategies or seek appropriate post-injury intervention [5,6]. However, ankle sprains can lead to serious consequences, including premature cessation of sports participation, especially in high-risk groups such as adolescent female volleyball players [7].

Lateral ankle sprains, which have a recurrence rate of up to 73%, may lead to the development of chronic ankle instability (CAI) [8,9,10]. CAI is defined by recurrent sprain episodes or sensations of the ankle “giving way”, and CAI is often accompanied by residual symptoms such as pain, muscle weakness, reduced ankle range of motion, and impaired ankle function [9,10]. Moreover, CAI may contribute to additional physical impairments by systematically affecting other joints [9]. It has also been associated with secondary injuries, such as post-traumatic ankle osteoarthritis and increased mechanical loading on the anterior cruciate ligament [8].

CAI, which is characterized by proprioceptive, neuromuscular, sensorimotor, and postural control deficits, can adversely affect athletes’ careers, long-term participation in sport, and overall well-being [1,9,11]. Although these negative effects of CAI have been well-documented in adult athletes, significantly less is currently known about its impact on adolescent athletes [7,12]. However, because adolescent athletes are still growing and developing their athletic skills [12], CAI may pose an even greater threat, making it critical to investigate its effects in this population.

Clarifying the impacts of CAI on adolescent athletes can guide healthcare professionals in developing intervention strategies, encourage athletes to adhere to post-injury rehabilitation, and motivate coaches to integrate preventive strategies into training programs.

To the best of the authors’ knowledge, the impacts of CAI on functional movement quality, dynamic balance, jumping performance, hip muscle strength, and injury risk among adolescent female volleyball players have not been previously investigated. Therefore, the primary purpose of this study was to determine the impact of CAI on functional movement quality by comparing adolescent female volleyball players with unilateral CAI (CAI group) and those without CAI (control group). The secondary purpose of this study was to compare the groups in terms of dynamic balance, vertical jumping performance, hip muscle strength and injury risk.

## 2. Materials and Methods

### 2.1. Study Design and Procedure

The present study was conducted at the Sports Physiotherapy Unit of the Faculty of Physical Therapy and Rehabilitation at Hacettepe University in Ankara, Turkey. We employed a cross-sectional comparative study design, which involves observational data at a single time point [13].

The study was performed in two sequential phases at a single time point. In the first phase of the study, all individuals were interviewed for descriptive data and thorough medical history, they completed the Turkish version of the Identification of Functional Ankle Instability (IdFAI) scale, and their dominant lower extremity was determined according to the procedure of Vauhnik et al. [14,15]. This phase of the study was carried out by the researcher Z.E. and sports physician A.M.A. In the second phase, all measurements were performed by the researcher A.S.A., who was unaware of which groups the individuals were in.

### 2.2. Individuals

Individuals were recruited from sports high schools in Ankara, Turkey. The sample of the study consists of adolescent female volleyball players (14–18 years of age) who have been training regularly (at least 3 days/week and 1 h/day) for the last year or longer and have competed in school athletics at least once a year.

In the first phase, individuals were initially screened for eligibility using the International Ankle Consortium inclusion and exclusion criteria for the CAI and control groups [10].

Individuals were included in the CAI group if they (1) had a history of at least 1 significant initial ankle sprain, and the initial sprain and the most recent sprain must have occurred more than 1 year and 3 months, respectively, prior to enrolment in the study; (2) experienced at least two episodes of an “ankle giving way” in the 6 months prior to enrolment in the study; and/or (3) had recurrent sprain (at least two sprains to the same ankle); and/or (4) had feelings of instability of the sprained ankle assessed with the IdFAI score ≥ 11 [10,14,16]. In addition, individuals with unilateral CAI only affecting the dominant limb were included in the CAI group. Furthermore, confirmation of ankle instability through a clinical examination by the sports physician (A.M.A.) was required to be included in the CAI group.

The inclusion criteria for the control group were as follows: (1) no history of ankle sprain to either ankle, (2) no history of an ankle giving way, and (3) having no feeling of instability for either ankle, as assessed using an IdFAI score ≤ 10 [14,16].

Individuals were excluded from the study if they (1) had a history of previous surgery or a fracture to the musculoskeletal structures of either lower extremity; (2) had an acute musculoskeletal injury of the lower extremity in the previous 3 months, disrupting their desired physical activity for at least 1 day; and (3) had any other health problems (such as neurological, rheumatologic, and vestibular diseases) that would affect the assessments of the study [10]. Additionally, individuals were also excluded from the study if they had CAI affecting the non-dominant ankle or both ankles and engaged in vigorous physical activity within the 24 h prior to assessments [17].

**Figure 1:**
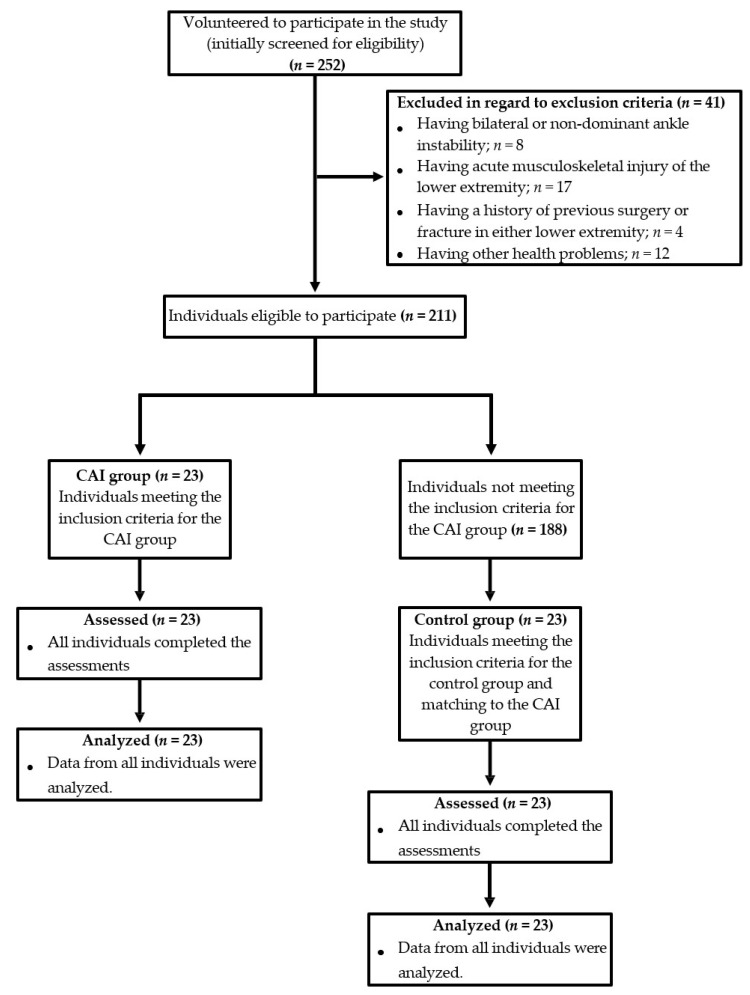
Flow diagram of the study.

Two hundred and fifty-two individuals and their parents agreed to participate in the study and signed the informed consent forms (280 invited/252 accepted). Based on the exclusion criteria, 41 of the 252 individuals (initially screened for eligibility) were excluded. Of the 211 individuals, 23 met the inclusion criteria for the CAI group (n = 23). Then, of the remaining 188 individuals, the control group was composed of an equal number of individuals (n = 23) who matched to the CAI group in terms of limb dominance, as well as ±10% in age, height, and weight [18,19]. All individuals (N = 46) completed the assessments without any missing data (assessed n = 46), and the data of all individuals were analyzed (analyzed n = 46). A flowchart of the study is shown in Figure 1.

In the study, a consecutive sampling method was used to obtain the required sample size. Therefore, individuals were included until the adequate statistical power was reached for the primary hypothesis (β ≥ 0.80 with α < 0.05), and a post hoc power was subsequently calculated [20].

### 2.3. Assessment Procedures and Measurement Tools

Individuals participated in a single assessment session at the end of the school sport season. The descriptive data included age, height, weight, and athletic history. As part of the medical history, the following details were recorded: date of the first significant ankle sprain, severity of the sprain(s), any history of treatment or rehabilitation, and frequency of recurrent sprains and episodes of an ankle giving way.

The IdFAI is a self-reported questionnaire specific to ankle instability, and the Turkish version of the questionnaire is valid and reliable for volleyball players [14]. Higher scores indicate more severe ankle instability, and the cut-off score was determined as ≥11 to identify ankle instability for the Turkish version of the IdFAI [14]. As there is no Turkish questionnaire assessing ankle instability for children, individuals completed the Turkish version of the IdFAI under the supervision of the researcher Z.E., who assisted them to comprehend the questions if necessary [21].

The clinical examination was conducted by sports physician A.M.A. (with 10 years of experience) and included special tests, such as the reverse anterolateral drawer test, anterolateral talar palpation, and traditional anterior drawer test [10,22].

In the second phase, the other assessments were systematically performed in accordance with previously described protocols in the literature by the blind assessor (the researcher A.S.A.) only.

All individuals were assessed in the same setting using the same measurement tools, in the following fixed order: (1) Functional Movement Screen (FMS), (2) Y-Balance Test (YBT), (3) vertical jumping performance, and (4) assessment of hip muscle strength. Individuals were given a 5 min rest between each assessment to minimize the effects of fatigue [18].

Each measurement was performed barefoot, with individuals wearing comfortable clothing. Before any measurements were taken, individuals completed a standardized warm-up consisting of 5 min of treadmill running at 6–10 km/h, followed by 5 min of static and dynamic stretching exercises [23,24].

For individuals in both groups, the YBT (in the posteromedial and posterolateral directions) and hip muscle strength were assessed on the dominant lower limb only. The YBT (in the anterior direction), FMS, and vertical jumping tests were performed bilaterally.

#### 2.3.1. Functional Movement Screen

The FMS was developed by Cook et al. to assess the quality of functional movements that require the integration of cognitive, perceptual, proprioceptive, and motor functions, and it has been comprehensively described elsewhere [25,26]. Furthermore, the FMS is used as a screening tool for injury prevention and performance predictability in athletic populations, owing to its ability to determine the level of functional movement competency [27]. The FMS is a valid and reliable tool for assessing movement patterns in youth volleyball players, and an FMS score of 14 or below has been identified as a significant risk factor for injury in this population [28].

The FMS consists of the following seven tests: (1) deep squat, (2) hurdle step, (3) in-line lunge, (4) shoulder mobility, (5) active straight leg raise, (6) trunk stability push-up, and (7) rotary stability. The total score of the FMS (FMS-composite score) ranges from 0 to 27 and a separate movement sub-score is calculated by adding together the scores on the deep squat, hurdle step, and in-line lunge (FMS-movement sub-score ranges from 0 to 9) [29].

The FMS was administered using the standardized FMS Test Kit (Functional Movement Systems, Inc., Chatham, VA, USA) in accordance with the standardized procedure [25,26]. Individuals were assessed, and their scores were systematically recorded by the researcher A.S.A., who has five years of clinical experience and is certified in FMS administration.

#### 2.3.2. Y-Balance Test

The YBT is a clinical assessment that measures dynamic neuromuscular control, balance, and physical performance, requiring strength, flexibility, proprioception, and coordination [30]. Additionally, it has been used to identify athletes at greater risk for lower-extremity injury, assess deficiencies following injury, and monitor rehabilitation progress [31].

The YBT has been established as a reliable measure of dynamic balance in adolescents and is commonly used in adolescent female volleyball players [31,32,33].

The YBTs were conducted according to previously described protocols using the Y-Balance Test Kit (Move2Perform, Evansville, IL, USA) [30,31]. While maintaining a single-leg stance, individuals were asked to reach with the free limb in the anterior (ANT), posteromedial (PM), and posterolateral (PL) directions in relation to the stance of the foot, keeping their hands on their hips [31]. Individuals practiced the six trials prior to formal testing. Before the first practice attempt, individuals were informed that any attempt involving the infractions, which are described in detail elsewhere [31,34], would not count.

When performing YBT, the standardized testing order was the right anterior, left anterior, dominant-side posteromedial, and dominant-side posterolateral. The YBT was performed only on the dominant lower limb in the PM and PL directions, while the ANT direction was assessed bilaterally. The absolute difference in the anterior-reach direction distance between limbs was calculated to assess side-to-side asymmetry [31,34]. It has been reported that an anterior asymmetry of more than or equal to 4 cm on the YBT has been linked to an increased risk of non-contact injury in Division I athletes [31,34].

Individuals completed three trials, and the greatest successful reach in each direction was recorded [30]. All reach distances were normalized to limb length, and a composite score was calculated as the average of the normalized reach distances [31]. Dominant limb length was measured from the anterior superior iliac spine (ASIS) to the medial malleolus using a cloth tape measure [30].

#### 2.3.3. Vertical Jump Tests

Jumping is a complex motor task that necessitates coordinated interaction between upper and lower body segments [35]. In volleyball players, vertical jump tests are considered valid and reliable measures for assessing the explosive power of the lower-extremity extensor muscles [36]. The heights of the squat jump with hands on the hips (SJwH), countermovement jump with hands on the hips (CMJwH), and countermovement jump with arm swing (CMJwA) were measured using the Optojump system (Microgate, Bolzano, Italy) [23,37].

The vertical jumping tests were conducted according to previously described procedures [23,35,37]. Following detailed instructions and demonstrations, individuals practiced the tests by performing submaximal vertical jumps for 5 min [23]. Then, 3 maximal jumps were performed for each test, and the highest result was recorded as the final result [35]. A rest interval of 30 s was provided between repetitions, and a 2 min rest was allowed between each series of jumps [23].

#### 2.3.4. Hip-Muscle Strength Measurements

The maximal isometric strength of the hip muscles (flexors, extensors, abductors, adductors, internal rotators, and external rotators) was measured using the “make test” method with a hand-held dynamometer (Lafayette Hand-Held Dynamometer, Model 01165; Lafayette Instrument Company, Indiana, USA) [38,39,40].

The validity and reliability of this assessment for hip muscles have been well established in adolescents, and it is commonly used in female youth athletes [38,39,41].

The measurements were performed according to previously described procedures, including individuals’ positioning, examiner positioning, dynamometer placement, verbal instructions, and applied stabilization [38,40]. Following detailed instructions and demonstrations, individuals practiced the measurement by performing 2 submaximal contractions. Then, the maximal isometric muscle contraction was sustained for 5 s, and the peak force was recorded in Newtons. Three trials were performed for each muscle group on the dominant leg, with a 30 s rest interval between each trial. The highest value was used for analysis [39].

To ensure greater reliability, the hip flexors, adductors, internal rotators and external rotators were measured according to the procedure defined by Cichanowski et al., while Krause et al.’s protocol was used for the remaining muscle groups [38,40]. The short-lever method was used to assess the hip extensors, while the long-lever method was used to assess the hip abductors [40].

Before measuring strength, limb length measurements were performed to calculate torque values [40]. While individuals were in a supine position, the length measurements were taken from the ASIS to the apex of the medial malleolus, from the ASIS to the medial joint line of the knee and from the medial joint line of the knee to the apex of the medial malleolus [40].

Estimated torque was calculated using the following formula [40]:

Torque (Nm) = Force (N) × Moment Arm (m)

The moment arm lengths were obtained by subtracting the corresponding distances based on the placement of the hand-held dynamometer [40]. Subsequently, the torque values were normalized to individuals’ body weight using the following formula [39]:

Normalized Torque Value (Nm/kg) = Torque (Nm)/Body Weight (kg)

### 2.4. Statistical Analysis 

The descriptive characteristics of the individuals are presented as the mean (standard deviations) and median (25th and 75th percentiles) for numerical data and as frequencies (percentages) for categorical data. The normal distribution of the numerical variables was tested using visual methods (histograms and probability plots) and analytical methods (Shapiro–Wilk test). Since the numerical variables were not normally distributed (*p* < 0.05 from the Shapiro–Wilk test), the Mann–Whitney U test was used to detect the group differences in these variables. Group comparisons for the categorical variables were performed using the chi-squared (χ^2^) test.

Only the data obtained from the dominant lower limbs of the individuals in both the CAI and control groups were analyzed to compare the groups for YBT scores and hip-muscle strength values. For variables that were assessed bilaterally but resulted in a single value per individual (FMS scores, YBT-anterior asymmetry, and vertical jumping scores), group comparisons were conducted based on these values.

To demonstrate the magnitude of between-group differences in each variable, effect size estimates were calculated as the correlation coefficients “*r*” (ranging from −1.00 to 1.00) for the Mann–Whitney U tests and were calculated as the “Φ” coefficients (ranging from 0.00 to 1.00) for the chi-squared (χ^2^) tests [42,43,44]. Additionally, 95% confidence intervals (95% CIs) for the effect size estimates were also calculated for both “ *r* “ and “Φ” values. If the 95% CI did not cross zero, then it was considered a group difference [42,43,44]. The *r* values of 0.10–0.29 indicate a small effect, 0.30–0.49 a moderate effect, 0.50–0.69 a large effect, and ≥0.70 a very large effect [44]. For Φ, values of 0.00–0.10 represent a negligible effect, 0.10–0.20 a weak effect, 0.20–0.40 a moderate effect, 0.40–0.60 a relatively strong effect, 0.60–0.80 a strong effect, and 0.80–1.00 a very strong effect [43].

Statistical analyses were conducted using SPSS version 26.0 (IBM Corp., Armonk, NY, USA) and Microsoft Excel version 2019 (Microsoft Corp., Richmond, WV, USA). The level of statistical significance for all inferential analyses was set at *p* < 0.05.

#### The Post Hoc Power Analysis

The primary hypothesis of this study was that there would be a difference in FMS-composite scores between the CAI and control groups, which was tested using the Mann–Whitney U test. The post hoc power analysis was conducted using R version 4.5.1 to determine the exact power of the study (using the “powertools” package and the “ranksum” function; R Core Team, 2025). This analysis revealed that the study had adequate power (β = 0.828 with α = 0.007) to detect a meaningful difference (β ≥ 0.80 with α < 0.05) for the primary hypothesis (n1 = 23, n.ratio = 1, *p* = 0.81, alpha = 0.007, power = NULL, sides = 2).

## 3. Results

The final analysis was based on data from 23 individuals with CAI in the dominant ankle (CAI group) and 23 individuals without CAI (control group). No individual had any missing data.

### 3.1. Characteristics of the Individuals

Descriptive data and the IdFAI scores of the groups are presented in Table 1. There were no significant differences between the CAI and control groups in age, body weight, height, body mass index, athletic history, and lower-limb dominance (*p* > 0.05); however, as expected, the CAI group had significantly greater ankle instability compared to the control group based on the IdFAI scores for the dominant ankle (*p* < 0.001), according to the results of the Mann–Whitney U test (Table 1).

### 3.2. Quality of Functional Movements and Injury Risk

The results of the FMS for both groups are shown in Table 2. The composite score and the movement sub-score of the FMS were lower in the CAI group compared to the control group (*p* = 0.007 and *p* = 0.001, respectively), with both differences supported by moderate effect sizes (*r* = −0.40 and *r* = −0.49, respectively). Additionally, there was a difference in the proportions of injury-risk classification between the groups (*p* = 0.022), supported by a moderate effect size (Φ = 0.34, 95% CI: [0.09–0.57]). Accordingly, the CAI group had a higher risk of injury than the control group, based on the established cut-off value for the FMS composite score.

### 3.3. Dynamic Balance and Injury Risk

The YBT results for both groups are presented in Table 3. Although the CAI group had lower scores on the YBT-ANT, YBT-PM, YBT-PL, and YBT-composite than the control group, only the YBT-ANT score was statistically significant (*p* = 0.004), supported by a moderate effect size (*r* = −0.42, 95% CI: [(−0.63)–(−0.15)]). While the YBT-anterior asymmetry scores were higher in the CAI group than the control group, the difference between groups was not statistically significant (*p* >0.05). However, the CAI group had a higher proportion of individuals classified as high injury risk based on the YBT-anterior asymmetry cut-off value (*p* = 0.001), with a relatively strong effect size (Φ = 0.50, 95% CI: [0.23–0.69]).

### 3.4. Vertical Jumping Performance

The results of the vertical jumping performance for both groups are presented in Table 4. There were no statistically significant differences between the CAI and control groups in the scores of the SJwH, CMJwH, and CMJwA (*p* > 0.05).

### 3.5. Hip Muscle Strength

Table 5 presents the results of the normalized torque values for the hip muscles in both groups. Statistically significant lower values were observed only in the hip flexors and the hip adductors (*p* = 0.007 and *p* = 0.044, respectively), supported by moderate effect sizes (*r* = −0.40; 95% CI: [(−0.62)–(−0.13)] and *r* = −0.31; [(−0.56)–(−0.01)], respectively). However, no statistically significant differences were found between the groups in the other hip muscle groups (extensors, abductors, internal rotators, and external rotators) (*p* > 0.05).

## 4. Discussion

To the best of our knowledge, this is the first study to examine whether adolescent female volleyball players with unilateral CAI have impaired functional movement quality and reduced physical performance in balance, vertical jumping, and hip strength, as well as an elevated risk of injury, compared to those without CAI.

In the present study, the CAI group was found to have lower FMS-composite and FMS-movement scores, reduced YBT-anterior performance, and strength deficits in the hip flexor and adductor muscles. Additionally, a higher proportion of the athletes in the CAI group were classified as being at high risk of injury based on both FMS-composite and YBT-anterior asymmetry cut-off scores [28,34]. However, no differences were observed between the groups in other YBT scores, vertical jumping performance, or the other hip muscle groups.

Most of the existing knowledge regarding the impacts of CAI on functional outcomes and muscle strength comes from studies conducted in adult populations. However, there remains a notable lack of research addressing these parameters in children or adolescents [7,12,45,46]. To date, only three studies have investigated the effects of CAI or a history of lateral ankle sprain(s) in children or adolescent athletes, examining outcomes such as static and dynamic balance, postural stability, functional performance, lower-limb strength, and foot alignment [45,46,47].

A recent study by Suphasubtrakul et al. examined children aged 7 to 12 with CAI, comparing their lower-limb strength and balance with those of copers [45]. Children with CAI demonstrated poorer performance in static and dynamic balance tests, including the single-leg stance and all directions of the Y-Balance Test. Although conducted in a preadolescent population, the findings offer valuable insight into the consequences of CAI during developmental years. While direct comparison to adolescent athletes is limited, the results support the notion that CAI adversely affects neuromuscular function in children.

Another relevant study by Maeda et al. investigated the relationship between CAI, foot alignment, and dynamic postural stability in adolescent athletes from various sports [47]. Although their sample differed from the present study in terms of age range (12–17 years) and included participants, the study demonstrated that adolescent athletes with CAI exhibited more rearfoot valgus and impaired postural control following jump landing. These findings are in line with the current study’s results, particularly regarding reduced dynamic balance performance, and further highlight the balance-related impairments associated with CAI in adolescent athletes.

The third and most closely related study, conducted by Ko et al., evaluated functional performance in adolescent soccer players with a history of lateral ankle sprain(s) using the Star Excursion Balance Test (SEBT) and the Single-Leg Hop Test (SLHT) [46]. The injured group showed lower scores in all SEBT directions and slower SLHT performance compared to uninjured peers. Although FMS was not assessed and the sample differed in sport type, the findings align with the present study in demonstrating neuromuscular and postural control impairments associated with CAI. Alongside the reduced anterior reach on the YBT and impaired movement quality on the FMS in our study, these results further support that CAI compromises functional performance across adolescent athletic populations.

The FMS was primarily used in the present study to assess functional movement quality in adolescent athletes with CAI [27,29]. Our results indicate that these athletes exhibit less efficient movements, especially in tasks requiring dynamic stability, neuromuscular coordination, and postural control. These impairments likely reflect underlying sensorimotor deficits, supporting the idea that CAI disrupts motor control strategies essential for safe and effective athletic performance [27,28,29]. Supporting this interpretation, a cross-sectional study involving 170 adult collegiate athletes reported that individuals with a history of injury or surgery demonstrated lower FMS-composite scores, further reinforcing the link between impaired movement quality and prior musculoskeletal dysfunction [48].

For dynamic balance, the CAI group showed lower anterior reach scores on the YBT and nonsignificant decreases in the posteromedial and posterolateral directions. The anterior direction is particularly sensitive to limitations in ankle dorsiflexion range of motion and sensorimotor control, both of which are commonly impaired in adult individuals with CAI [49,50]. Our findings align with previous studies using the YBT or the SEBT, which have similarly reported directional deficits in individuals with CAI [45,49,50]. Taken together, the results suggest that anterior reach performance may serve as a more prominent and sensitive indicator of dynamic balance impairments in adolescent athletes with CAI.

In vertical jumping performance, although CAI is associated with neuromuscular deficits, no difference was found between groups. This finding is consistent with previous research indicating that individuals with CAI may report greater perceived disability yet demonstrate comparable performance in explosive lower-limb tasks [51]. Since vertical jump tests primarily assess concentric power rather than sensorimotor control, they may be less sensitive to functional impairments associated with CAI [36,52]. Consistent with this view, a recent meta-analysis reported that hop tests involving lateral movement are more effective than vertical jump tasks in detecting functional deficits [53]. Therefore, while CAI may impair balance and postural control, it does not appear to significantly affect vertical explosive power in adolescent female volleyball players.

For hip muscle strength, the present study revealed reductions in hip flexor and adductor strength in the CAI group, with no differences observed in the other muscle groups. These findings are consistent with previous research reporting selective hip muscle weakness in individuals with CAI. While some studies found no differences in hip abductors or extensors [54], others identified deficits in hip external rotators and abductors [49,55]. It is notable that hip strength, particularly in the frontal and transverse planes, has been linked to dynamic balance performance [49,56] and reduced gluteal activation during balance tasks in individuals with CAI further supports the presence of neuromuscular impairments [57].

Injury risk is a major concern for adolescent athletes due to neuromuscular asymmetries, postural control deficits, and the demands of asymmetrical sport-specific movements [58,59]. This risk is further amplified during adolescence, a critical period of neuromuscular development associated with increased susceptibility to musculoskeletal injuries [59]. In the present study, a larger proportion of athletes in the CAI group were classified as high risk for injury based on established cut-off values for the FMS-composite and YBT-anterior reach asymmetry, both of which are validated indicators of injury risk in youth athletes [28,34]. Our findings are supported by previous studies. Letafatkar et al. demonstrated that individuals with lower FMS scores had a significantly higher risk of lower-extremity injuries, and Chimera et al. found that collegiate athletes with a history of injury exhibited lower FMS and YBT scores [48,60]. These results confirm our finding that adolescent athletes with CAI have a higher risk of injury.

Ankle injuries are common among adolescent athletes and can lead to serious consequences [1,3,7,9,11,61]. The present study is considered to fill an important gap in the literature by revealing the effects of CAI in this population. Unlike previous research, which has primarily focused on adult athletes, our study used a robust methodology to demonstrate the impact of CAI on movement quality, dynamic balance, hip muscle strength, and injury risk in adolescent female volleyball players.

In the present study, although statistically significant group differences supported by moderate-to-large effect sizes were detected in some variables, the group differences in means or medians were relatively small. Therefore, the clinical relevance of these findings should be interpreted with caution. These differences can represent functional deficits in adolescent athletes with CAI. However, as there are no studies that establish the minimal clinically important difference values of the assessment tools used in our study for this population, the clinical significance of our findings may not be definitively clarified. Nevertheless, in adolescent athletes, even deficits that do not reach a clinically significant threshold could still predispose them to injury or negatively affect their developing athletic performance. Moreover, these deficits may worsen over time. In this context, addressing even small functional deficits caused by CAI may help prevent injuries and enhance athletic performance.

This study has several limitations. First, the sample consisted solely of female volleyball players recruited from a single geographic region (Ankara, Turkey), which restricts the generalizability of the findings to other adolescent volleyball players, particularly those from different sexes, regions, or competitive levels. The main reason for including only female individuals was that sex has been shown in the literature to act as a covariate influencing the outcomes of functional assessment tests [48,62]. Another limitation is the lack of a Turkish-validated instrument specifically designed to assess ankle instability in children. For this reason, individuals completed the Turkish version of the IdFAI under the supervision of the researcher, who provided clarification when necessary [12]. Additionally, including prospective follow-up in the present study would have provided clearer evidence of the causal relationship between CAI and injury risk. Moreover, if the present study had included a larger CAI group with different CAI severity, subgroup analyses could have revealed a potential dose–response relationship. Finally, objective laboratory-based technologies such as three-dimensional motion analysis systems were not employed in this study. Instead, the effects of CAI were evaluated using valid and reliable functional assessments that can be practically applied in accessible clinical settings [53,58].

## 5. Conclusions

Our results suggest that adolescent female volleyball players with CAI show deficits in neuromuscular control, dynamic balance, and hip muscle strength, which can affect athletic performance and increase vulnerability to future injuries. The study may raise awareness among athletes and coaches about the potentially serious consequences of ankle injuries, thereby supporting the long-term athletic development and overall health of adolescent athletes.

It is anticipated that our findings will guide intervention strategies that incorporate specific hip-strengthening exercises (especially for the flexors and adductors), dynamic balance training (especially focused on anterior reach and single-leg stability), and neuromuscular control exercises to enhance movement quality. In this high-risk population, integrating these components into regular training routines may help improve functional outcomes and reduce the risk of injury associated with CAI.

Future research should focus on larger, more diverse samples of adolescent athletes to increase the generalizability of findings and improve understanding of the underlying mechanisms of CAI-caused deficits. Additionally, the investigation of effective interventions aimed at optimizing performance and reducing the risk of re-injury in adolescents with CAI is warranted.

## Figures and Tables

**Table 1:** Descriptive characteristics of the CAI and control groups.

	CAI Group (*n* = 23)		Control Group (*n* = 23)		*p*-Value
	X (SD)	M (25–75%)	X (SD)	M (25–75%)	
Age (year)	14.91 (0.9)	15 (14–15)	14.96 (0.9)	15 (14–16)	*p*^a^ = 0.796
Body weight (kg)	63.54 (8.1)	63 (59–71)	58.64 (7.4)	59.1 (54–63)	*p*^a^ = 0.062
Height (cm)	174.03 (7.5)	174 (170–177.7)	170.85 (8.5)	172 (164–177)	*p*^a^ = 0.191
Body mass index (kg/m^2^)	20.99 (2.5)	20.86 (19.4–22.5)	20.03 (1.5)	19.79 (19.4–21.1)	*p*^a^ = 0.177
Starting age of volleyball specific training (year)	10.57 (1.5)	11 (10–12)	10.09 (1.7)	10 (9–11)	*p*^a^ = 0.215
IdFAI score of the dominant lower limb (0–37)	15.5 (5.2)	14 (11–18)	4.9 (3.3)	5 (1.5–7.5)	*p*^a^ < 0.001 *
	***n* (%)**		***n* (%)**		
Lower-limb dominance					*p*^b^ = 1.0
Right	15 (65.2)		15 (65.2)		
Left	8 (34.8)		8 (34.8)		

X: mean; SD: standard deviation; M: median; 25–75%: 25th and 75th percentiles; kg: kilogram; cm: centimeter; kg/m^2^: kilogram per square meter; IdFAI: Identification of Functional Ankle Instability; *p*^a^: *p*-value from the Mann–Whitney U test; *p*^b^: *p*-value from the chi-squared (Χ^2^) test. * Significance level of *p* < 0.05.

**Table 2:** Results of the FMS for the CAI and control groups.

	CAI Group (*n* = 23)		Control Group (*n* = 23)		*p*-Value		Effect Size [95% CI]
	X (SD)	M (25–75%)	X (SD)	M (25–75%)		Ζ	*r*
Composite FMS score (0–27)	15 (2)	15 (13–17)	16.65 (1.6)	17 (16–18)	*p*^a^ = 0.007 *	−2.69	−0.40 [(−0.62)–(−0.13)]
Movement sub-score of the FMS (0–9)	6.57 (1.2)	7 (6–7)	7.78 (0.9)	8 (7–8)	*p*^a^ = 0.001 *	−3.32	−0.49 [(−0.69)–(−0.23)]
	***n* (%)**		***n* (%)**			**Χ^2^**	**Φ**
Composite FMS score					*p*^b^ = 0.022 *	5.25	0.34 [(0.09)–(0.57)]
≤14	10 (43.5)		3 (13.1)				
>14	13 (56.5)		20 (86.9)				

FMS: Functional Movement Screen; 95% CI: 95% confidence interval for the effect size; X: mean; SD: standard deviation; M: median; 25–75%: 25th and 75th percentiles; Z: standardized Z-score from the Mann–Whitney U test; *r*: correlation coefficient for the effect size; Χ^2^: chi-square statistic from the chi-squared (Χ^2^) test; Φ: phi coefficient for the effect size; *p*^a^: *p*-value from the Mann–Whitney U test; *p*^b^: *p*-value from the chi-squared (Χ^2^) test. * Significance level of *p* < 0.05.

**Table 3:** Results of the YBT for the CAI and control groups.

	CAI Group (*n* = 23)		Control Group (*n* = 23)		*p*-Value		Effect Size [95% CI]
	X (SD)	M (25–75%)	X (SD)	M (25–75%)		Ζ	*r*
YBT-Anterior (%)	67.6 (12.9)	74.1 (55.5–78.8)	76.9 (14.1)	84.6 (61.1–89.1)	*p*^a^ = 0.004 *	−2.87	−0.42 [(−0.63)–(−0.15)]
YBT-Posteromedial (%)	74.8 (18)	78.9 (60.8–92)	83.6 (12.9)	84.9 (74.3–90.5)	*p*^a^ = 0.147	−1.45	−0.21 [(−0.47)–(0.07)]
YBT-Posterolateral (%)	79.8 (17.7)	79.6 (63.9–96)	87 (13.7)	90.2 (69.3–97.6)	*p*^a^ = 0.170	−1.37	−0.20 [(−0.46)–(0.08)]
YBT-Composite (%)	74.1 (15.7)	79.3 (58.2–89.7)	82.5 (12.8)	87.5 (68.4–92.5)	*p*^a^ = 0.057	−1.90	−0.28 [(−0.54)–(0.02)]
YBT-Anterior asymmetry (cm)	4.9 (4)	3.5 (1.2–9.6)	2.2 (1.2)	2.3 (1.3–3)	*p*^a^ = 0.073	−1.79	−0.26 [(−0.52)–(0.03)]
	***n* (%)**		***n* (%)**			**Χ^2^**	**Φ**
YBT-Anterior asymmetry					*p*^b^ = 0.001 *	11.28	0.50 [(0.23)–(0.69)]
≥4 cm	11 (47.8)		1 (4.4)				
<4 cm	12 (52.2)		22 (95.6)				

YBT: Y-Balance Test; cm: centimeter; 95% CI: 95% confidence interval for the effect size; X: mean; SD: standard deviation; M: median; 25–75%: 25th and 75th percentiles; Z: standardized Z-score from the Mann–Whitney U test; *r*: correlation coefficient for the effect size; Χ^2^: chi-square statistic from the chi-squared (Χ^2^) test; Φ: phi coefficient for the effect size; *p*^a^: *p*-value from the Mann–Whitney U test; *p*^b^: *p*-value from the chi-squared (Χ^2^) test. * Significance level of *p* < 0.05.

**Table 4:** Results of the vertical jumping performance for the CAI and control groups.

	CAI Group (*n* = 23)		Control Group (*n* = 23)		*p*-Value		Effect Size [95% CI]
	X (SD)	M (25–75%)	X (SD)	M (25–75%)		Ζ	*r*
SJwH (cm)	22.6 (3.4)	23.1 (20.3–25)	22.7 (3.2)	22.8 (20.7–25)	*p*^a^ = 0.843	−0.20	−0.03 [(−0.32)–(0.26)]
CMJwH (cm)	24.9 (3.1)	25 (23.1–27.5)	24.3 (3.2)	24.4 (22.1–26.6)	*p*^a^ = 0.455	−0.75	−0.11 [(−0.38)–(0.17)]
CMJwA (cm)	29.4 (3.9)	29.1 (26.5–31.9)	28.9 (3.5)	28.5 (26.1–31.3)	*p*^a^ = 0.503	−0.67	−0.10 [(−0.37)–(0.18)]

SJwH: squat jump with hands on the hips; CMJwH: counter movement jump with hands on the hips; CMJwA: counter movement jump with arm swing; cm: centimeter; 95% CI: 95% confidence interval for the effect size; X: mean; SD: standard deviation; M: median; 25–75%: 25th and 75th percentiles; Z: standardized Z-score from the Mann–Whitney U test; *r*: correlation coefficient for the effect size; *p*^a^: *p*-value from the Mann–Whitney U test.

**Table 5:** Hip muscles torque values for the CAI and control groups, normalized to body weight.

	CAI Group (*n* = 23)		Control Group (*n* = 23)		*p*-Value		Effect Size [95% CI]
	X (SD)	M (25–75%)	X (SD)	M (25–75%)		Ζ	*r*
Hip flexors (Nm/kg)	1.40 (0.31)	1.38 (1.19–1.64)	1.63 (0.21)	1.62 (1.52–1.77)	*p*^a^ = 0.007 *	−2.69	−0.40 [(−0.62)–(−0.13)]
Hip extensors (Nm/kg)	2.38 (0.35)	2.44 (2.12–2.49)	2.59 (0.35)	2.62 (2.32–2.76)	*p*^a^ = 0.062	−1.87	−0.28 [(−0.54)–(0.02)]
Hip abductors (Nm/kg)	2.08 (0.67)	2.13 (1.54–2.51)	2.26 (0.40)	2.24 (1.92–2.59)	*p*^a^ = 0.253	−1.14	−0.17 [(−0.44)–(0.11)]
Hip adductors (Nm/kg)	1.12 (0.28)	1.11 (0.88–1.36)	1.26 (0.17)	1.25 (1.16–1.34)	*p*^a^ = 0.044 *	−2.11	−0.31 [(−0.56)–(−0.01)]
Hip internal rotators (Nm/kg)	0.74 (0.13)	0.73 (0.66–0.83)	0.75 (0.14)	0.75 (0.66–0.88)	*p*^a^ = 0.965	−0.04	−0.01 [(−0.29)–(0.28)]
Hip external rotators (Nm/kg)	0.59 (0.07)	0.60 (0.55–0.64)	0.60 (0.09)	0.61 (0.55–0.66)	*p*^a^ = 0.886	−0.14	−0.02 [(−0.30)–(0.27)]

Nm/kg: Newton-meters per kilogram; 95% CI: 95% confidence interval for the effect size; X: mean; SD: standard deviation; M: median; 25–75%: 25th and 75th percentiles; Z: standardized Z-score from the Mann–Whitney U test; *r*: correlation coefficient for the effect size; *p*^a^: *p*-value from the Mann–Whitney U test. * Significance level of *p* < 0.05.

## Data Availability

The raw data supporting the conclusions of this article will be made available by the authors upon request.

## Abbreviations

The following abbreviations are used in this manuscript:

CAI	Chronic ankle instability
FMS	Functional movement screen
YBT	Y-Balance Test
IdFAI	Identification of functional ankle instability
ANT	Y-Balance Test—anterior direction
PM	Y-Balance Test—posteromedial direction
PL	Y-Balance Test—posterolateral direction
SJwH	Squat jump with hands on the hips
CMJwH	Countermovement jump with hands on the hips
CMJwA	Countermovement jump with arm swing
ASIS	Anterior superior iliac spine
SPSS	Statistical Package for the Social Sciences
CI	Confidence interval
SEBT	Star Excursion Balance Test
SLHT	Single-Leg Hop Test
